# Computational Methods to Study Human Transcript Variants in COVID-19 Infected Lung Cancer Cells

**DOI:** 10.3390/ijms22189684

**Published:** 2021-09-07

**Authors:** Jiao Sun, Naima Ahmed Fahmi, Heba Nassereddeen, Sze Cheng, Irene Martinez, Deliang Fan, Jeongsik Yong, Wei Zhang

**Affiliations:** 1 Department of Computer Science, University of Central Florida, Orlando, FL 32816, USA; jiao.sun@knights.ucf.edu (J.S.); fnaima@knights.ucf.edu (N.A.F.); 2 Genomics and Bioinformatics Cluster, University of Central Florida, Orlando, FL 32816, USA; hebanasser@knights.ucf.edu; 3 Department of Electrical and Computer Engineering, University of Central Florida, Orlando, FL 32816, USA; 4 Department of Biochemistry, Molecular Biology and Biophysics, University of Minnesota Twin Cities, Minneapolis, MN 55455, USA; cheng705@umn.edu; 5 Department of Molecular Biotechnology, Universität Heidelberg, 69120 Heidelberg, Germany; irene.martinez@stud.uni-heidelberg.de; 6 School of Electrical, Computer and Energy Engineering, Arizona State University, Tempe, AZ 85287, USA; dfan@asu.edu

**Keywords:** COVID-19, transcript variants, alternative splicing, alternative polyadenylation, RNA-seq, 3′-UTR

## Abstract

Microbes and viruses are known to alter host transcriptomes by means of infection. In light of recent challenges posed by the COVID-19 pandemic, a deeper understanding of the disease at the transcriptome level is needed. However, research about transcriptome reprogramming by post-transcriptional regulation is very limited. In this study, computational methods developed by our lab were applied to RNA-seq data to detect transcript variants (i.e., alternative splicing (AS) and alternative polyadenylation (APA) events). The RNA-seq data were obtained from a publicly available source, and they consist of mock-treated and SARS-CoV-2 infected (COVID-19) lung alveolar (A549) cells. Data analysis results show that more AS events are found in SARS-CoV-2 infected cells than in mock-treated cells, whereas fewer APA events are detected in SARS-CoV-2 infected cells. A combination of conventional differential gene expression analysis and transcript variants analysis revealed that most of the genes with transcript variants are not differentially expressed. This indicates that no strong correlation exists between differential gene expression and the AS/APA events in the mock-treated or SARS-CoV-2 infected samples. These genes with transcript variants can be applied as another layer of molecular signatures for COVID-19 studies. In addition, the transcript variants are enriched in important biological pathways that were not detected in the studies that only focused on differential gene expression analysis. Therefore, the pathways may lead to new molecular mechanisms of SARS-CoV-2 pathogenesis.

## 1. Introduction

Recent research on the severe acute respiratory syndrome coronavirus 2 (SARS-CoV-2) has focused primarily on gene expression profiling in various human cell lines and patient samples in an effort to understand the molecular basis of SARS-CoV-2 pathogenesis [1,2,3]. Unsurprisingly, many of the differentially expressed host genes identified are involved in the antiviral response [1,4]. As the SARS-CoV-2 virus can trigger different levels of changes in humans, a thorough understanding of molecular signatures beyond gene expression profiling is needed to comprehensively dissect the pathogenesis of SARS-CoV-2 coronavirus.

Immune activation to viral infection is regulated by changes in gene expression and post-transcriptional regulation, such as AS and APA [5,6]. A classic example of AS in immune cells is the generation of neoantigens, novel peptides of immune proteins for antibody diversification in normal conditions [7]. However, during viral infection by the influenza A virus, the virus can induce widespread AS in the host genes [5,7,8]. Mechanically, viral proteins can interact with splicing and polyadenylation factors and cause misprocessing of the host mRNAs [8]. Some of these isoforms may encode unique neoantigens or disrupt critical functional domains, thereby promoting viral replication and production [7,8,9]. These studies stress the importance of the RNA-processing regulation upon viral infection. A recent study suggests that the NSP16 viral protein of SARS-CoV-2 can interact with the human U1 and U2 snRNA, thereby changing the AS landscape in humans [10]. This warranted our investigation of the host splicing alteration during SARS-CoV-2 infection to further reveal the molecular basis of the COVID-19 disease.

**Figure 1:**
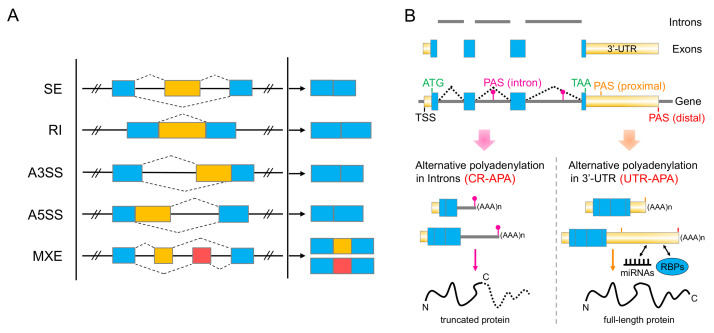
Alternative splicing (AS) and alternative polyadenylation (APA). (**A**) Schematic representation of the five major types of alternative splicing in eukaryotes: Skipped Exon (SE), Retained Intron (RI), Alternative 3${}^{\prime }$ Splice Site (A3SS), Alternative 5${}^{\prime }$ Splice Site (A5SS), and Mutually Exclusive Exon (MXE). Light blue boxes represent the constitutive exons, whereas light yellow and pink exons represent the spliced ones. (**B**) Two types of alternative polyadenylation, i.e., coding region alternative polyadenylation (CR-APA) and 3${}^{\prime }$-UTR alternative polyadenylation (UTR-APA), and their impact on the functional proteome. PAS: polyadenylation signal; TSS: transcription start site; RBP: RNA-binding protein.

Here, RNA-seq data of SARS-CoV-2 infected lung cancer (A549) cells are used to investigate the SARS-CoV-2-inducted remodeling of the host transcriptome [1]. By utilizing computational methods and pipelines, including custom-developed tools, we have identified global AS and APA (Figure 1) changes upon SARS-CoV-2 infection in human lung epithelial cells. These widespread splicing signatures rarely overlap with findings from differential gene expression studies and are enriched in many previously undetected pathways critical for SARS-CoV-2 pathobiology.

## 2. Results

In this study, multiple bioinformatics pipelines and statistical methods were used to identify the molecular signatures of COVID-19 at the transcriptional and post-transcriptional levels. The objective is to achieve a better understanding of the impact of transcriptomic changes on the proteome in COVID-19. In this section, we first describe previously published RNA-seq data of COVID-19 [1] that are used in this study (Section 2.1). Then in the following subsections, we focus on reporting the results of our analyses on transcriptome changes using these datasets: we will extensively discuss the alterations of splicing and polyadenylation events as well as the differential expression of genes/transcripts upon SARS-CoV-2 infection in A549 human lung cancer cells.

### 2.1. Data

The RNA-seq data used in this study were downloaded from the NCBI Gene Expression Omnibus (GEO) database, under the accession number GSE147507 [1]. The dataset contains independent biological triplicates that were mock-treated or infected with SARS-CoV-2. Series 2, 5, and 7 in the dataset were analyzed in this study. Series 2 and 5 consist of transformed lung alveolar (A549) samples that are mock-treated or infected with SARS-CoV-2. Series 7 includes samples from transformed lung-derived Calu-3 cells that are mock-treated or infected with SARS-CoV-2.

We used TopHat2 [11] to conduct sequence alignment. These three pairs of cell line data (Series 2, 5, and 7) with a high overall alignment rate were selected for downstream analyses (Supplementary Table S1). Series 5 results are discussed in this manuscript, whereas results for the other two series can be found in the Supplementary Materials (Supplementary Tables S2–S8). The RNA-seq data were quantified with Kallisto [12] for differential transcript and gene expression analyses. To generate the read coverage profile, SAMtools [13] was used with the aligned bam files as an input.

### 2.2. Alternative Splicing

Five major AS events between groups of mock-treated and SARS-CoV-2 infected A549 cells are reported in Table 1. The table displays information regarding the number of AS events in each category. Overall, more AS events were detected in SARS-CoV-2 infected cells than in mock-treated ones. Among the five types of AS events, the Skipped Exon (SE) category was found to be the most common type; 51 AS events were identified in the mock-treated cells, and 126 AS events were identified in the SARS-CoV-2 infected cells. Notably, compared to other types of AS events, the AS event for alternative 5’ splice site (A5SS) was more frequent in the SARS-CoV-2 infected cells. We further investigated the top 10 significant events for the AS type SE among the two groups (Table 2). The AS events are sorted in ascending order by the *p*-value, as defined in the Chi-squared hypothesis test. The column ‘Ratio Difference’ denotes the differences in the ratio of average read coverage between the SARS-CoV-2 infected and mock-treated cells. A positive value for the ratio difference indicates that the mock-treated cells have more SE splicing events compared to the SARS-CoV-2 infected cells, and a negative value means the other way around.

**Figure 2:**
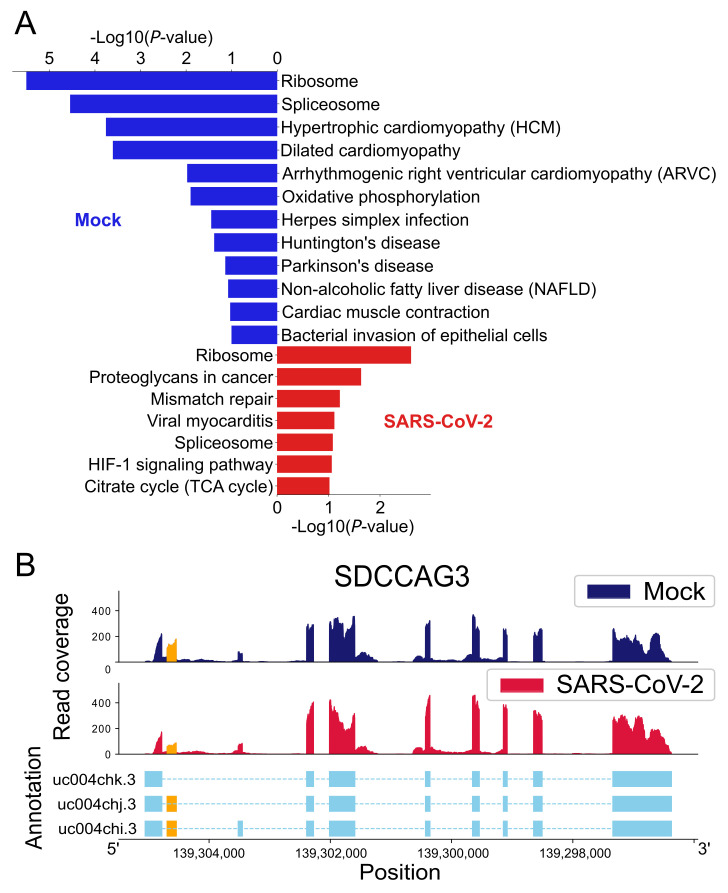
(**A**) KEGG pathways enriched by alternative splicing events. The blue and red bar charts show the pathways enriched by the splicing events in mock-treated cells and SARS-CoV-2 infected cells, respectively. (**B**) A plot for the *SDCCAG3* AS event. The spliced exon is highlighted in orange. The first two subplots show the read coverage of the gene in both groups, whereas the bottom subplot denotes the gene annotation with exon information. The *x*-axis and *y*-axis of the plot represent the position of the specific gene and read coverage of that sample, respectively. To show the differential splicing event, the altered exon is highlighted in orange to provide a clear insight into the phenomenon.

From the differential AS analysis performed in Table 1, we conducted a literature survey of the genes with significant AS events. Three of them are implicated in SARS-CoV-2 pathogenesis and prognosis as shown in Table 3. In addition, the genes with the splicing events were analyzed with the DAVID functional annotation tool [14]. The enriched KEGG pathways in mock-treated and SARS-CoV-2 infected cells are shown in Figure 2A. Interestingly, genes with AS events are enriched in ribosome and spliceosome, suggesting that SARS-CoV-2 infected cells undergo changes in their gene expression pathways.

Figure 2B shows a high resolution read coverage plot generated by our AS-Quant pipeline [26] for the gene *SDCCAG3* with a significant AS event. The figure illustrates the notable alternation of expression levels in the spliced exons between the mock-treated and SARS-Cov-2 infected groups.

### 2.3. Alternative Polyadenylation

To further assess the predictions of alternative polyadenylation events between mock-treated and the SARS-CoV-2 infected cells, both CR-APA and UTR-APA events (Figure 1B) were identified by the computational pipelines introduced in Section 4.2.

#### 2.3.1. UTR-APA

**Figure 3:**
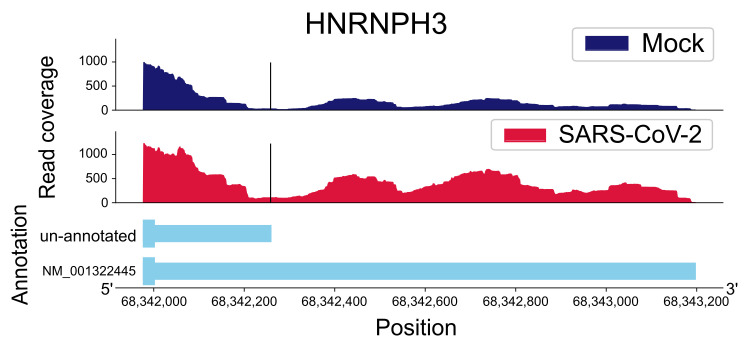
One example of a gene (*HNRNPH3*) shows an alternative polyadenylation event in the 3${}^{\prime }$-UTR region. The black vertical line indicates the potential cleavage site. The *x*-axis and *y*-axis represent the position of the gene in its chromosome and the read coverage, respectively. The top two subplots show the changes in read coverage surrounding the event, and the cleavage site is indicated by a black vertical line. The bottom part of the plot shows both the full length and truncated 3${}^{\prime }$-UTRs.

Our pipeline, APA-Scan [27], identified 144 unannotated UTR-APA events, which were reported using the Chi-squared hypothesis test on A549 cells. A total of 137 UTR-APA events were identified in mock-treated cells, while only 7 UTR-APA events were found in SARS-CoV-2 infected cells, indicating that UTR-APA events were suppressed in SARS-CoV-2 infected cells. Figure 3 shows a UTR-APA event for the gene *HNRNPH3* generated by APA-Scan. In Table 4, the top 10 UTR-APA events are reported by the *p*-value in the ascending order. The *p*-values indicate the significance of the UTR-APA event between mock versus SARS-CoV-2 infected cells. The ratio difference in the last column is calculated by taking the average read coverage ratios of the two groups. A positive ratio difference indicates an increase in the UTR-APA event in mock-treated cells. Based on the results reported in Table 4, all the top 10 events were identified from the mock-treated cells, thereby reinforcing that SARS-CoV-2 infections suppress the usage of the proximal polyadenylation signal in the 3${}^{\prime }$-end processing of mRNAs. The literature survey shows that three genes (*ANXA2*, *CAV1*, and *TMEM97*) with the UTR-APA events related to SARS-CoV-2 pathogenesis and prognosis (Table 3). For example, TMEM97 has been recently shown to interact with SARS-CoV-2 viral proteins [20].

#### 2.3.2. CR-APA

**Figure 4:**
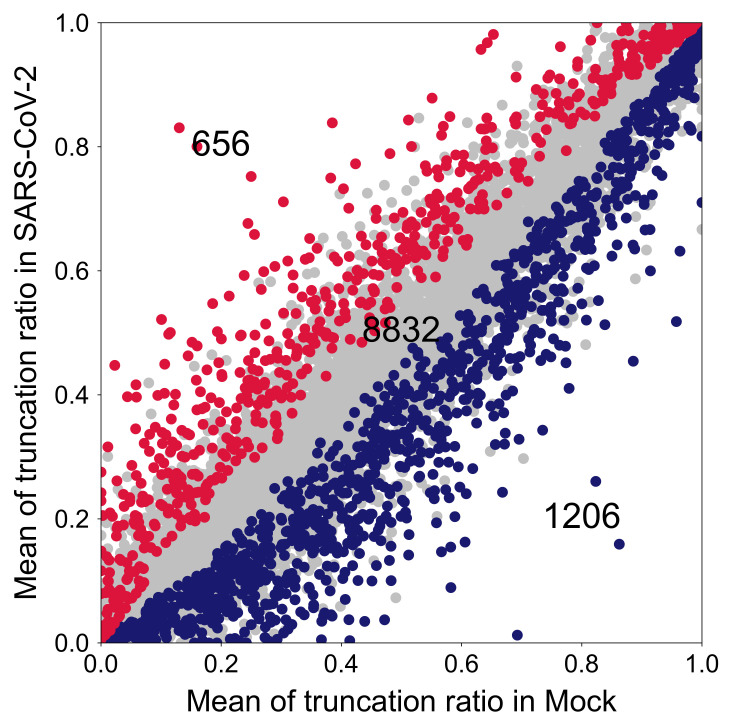
RNA-seq data of mock-treated and SARS-CoV-2 infected A549 cells were analyzed for the CR-APA. The *x*-axis and *y*-axis represent the CR-truncation ratios ((short mRNA)/(total mRNA)) of a gene. Each dot represents a gene, where the blue dots and red dots show the significant ones in mock and SARS-CoV-2 infected cells, respectively. Upon SARS-CoV-2 infection, 656 genes showed up-regulated CR-APA while 1206 genes showed down-regulated CR-APA. A total of 8832 genes remained unchanged in their CR-APA.

In the CR-APA analysis, both the hg38 RefSeq annotation [28] and the UCSC annotation [29] were applied to report the list of CR-APA events between mock-treated versus SARS-CoV-2 infected cells. We identified significant CR-APA events by measuring the CR-truncation ratio (Equation (4)). We found an increase of 1206 CR-APA events in mock-treated cells and an enrichment of 656 CR-APA events in SARS-CoV-2 infected cells, indicating that CR-APA events are more frequent in mock-treated cells. The bipartite expression patterns of CR-APA in Figure 4 revealed an enrichment of specific truncated mRNAs in both mock and SARS-CoV-2 infected cells, indicating that reprogramming of the transcriptome and consequent changes in the proteome diversity would occur upon SARS-CoV-2 infection.

**Figure 5:**
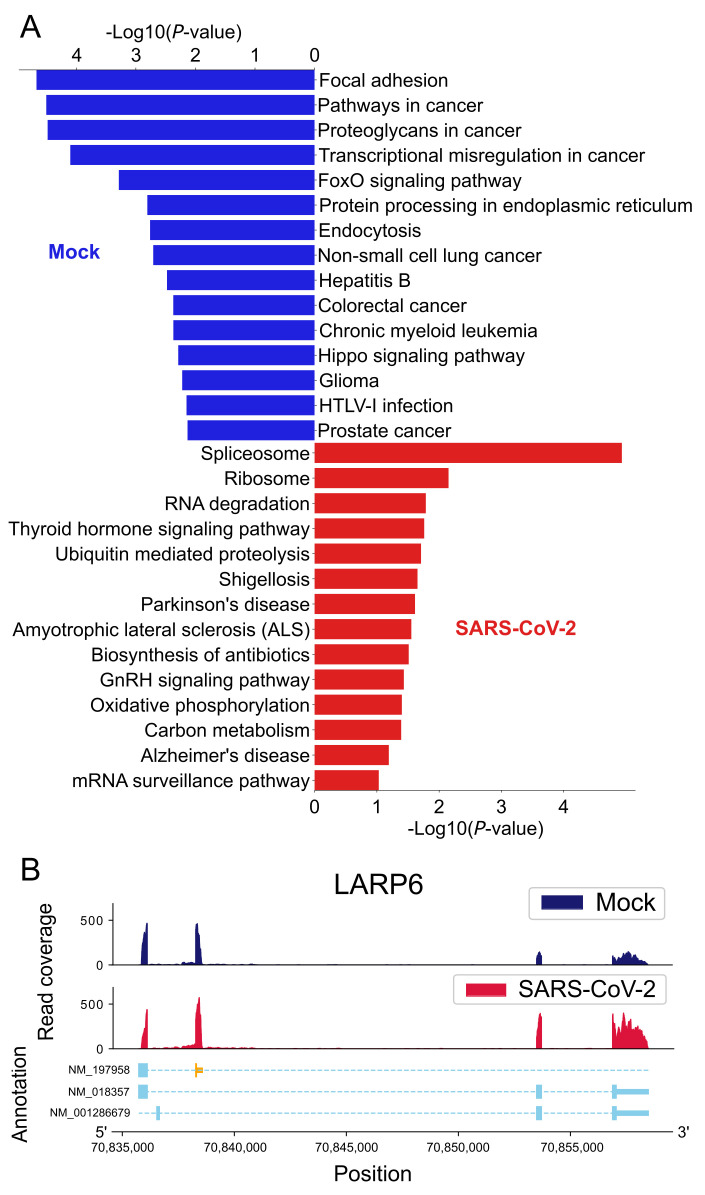
(**A**) KEGG pathways enriched by CR-APA events. The blue and red bar charts show the pathways enriched by the APA events in mock-treated cells and SARS-CoV-2 infected cells, respectively. (**B**) One example of a gene (*LARP6*) shows an alternative polyadenylation event in the coding region. The truncated coding exon is highlighted in orange.

Among the significant CR-APA events, the top 10 significant events are listed in Table 5. The ratio difference is calculated by taking the difference between the average CR-truncation ratio of the SARS-CoV-2 infected cells and mock-treated cells. A CR-APA event occurs in SARS-CoV-2 infected samples if the ratio difference is larger than zero. It is considered significant in the mock-treated group if the ratio difference is smaller than zero. A literature review shows that several genes with CR-APA events are connected to SARS-CoV-2 pathogenesis and prognosis, as described in Table 3. The KEGG pathways in mock-treated and the SARS-Cov-2 infected cells are shown in Figure 5A. Particularly, genes involved in oxidative phosphorylation increased CR-APA, which may affect the expression of full-length proteins that are critical for cellular energy production and mitochondrial dysfunction in respiratory illnesses [30]. It was shown that one of the SARS-CoV-2 proteins, NSP16, binds to the mRNA recognition domains of the spliceosome and suppresses global mRNA splicing in SARS-CoV-2-infected human cells [10]. The KEGG pathways enriched in Figure 2A and Figure 5A show AS and CR-APA events in genes associated with the spliceosome pathway, suggesting that alterations in the regulatory splicing pathway could happen. This, in turn, would affect splicing and splicing-coupled CR-APA events upon SARS-CoV-2 infection, leading to further deleterious effects on gene function [31].

Figure 5B shows a CR-APA event for the gene *LARP6*. The top two subplots show the read coverage of the gene in two conditions. The bottom panel represents the isoform annotations. NM_197958 is an annotated CR-truncated isoform in the gene, and the last highlighted exon indicates the end of the CR-APA transcript. Compared to SARS-CoV-2 infected cells, the plot shows a significant increase of RNA-seq alignments in the highlighted exon, indicating a higher CR-truncation ratio of *LARP6* in mock-treated cells compared to SARS-CoV-2 infected cells.

### 2.4. Differential Gene/Transcript Expression

**Figure 6:**
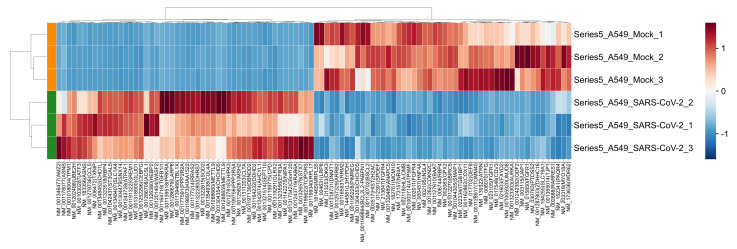
COVID-19 A549 cell lines are clustered by the top 100 transcript markers detected by differential transcript expression analysis.

In differential expression analysis, 991 genes and 1443 transcripts show higher expression in mock-treated cells, whereas 717 genes and 933 transcripts show higher expression in SARS-CoV-2 infected cells. These differentially expressed genes and transcripts can be served as molecular signatures for COVID-19 outcome prediction. The top 100 differentially expressed transcripts and genes were selected for bi-clustering analysis. The z-score transformation was applied to the expression value for each entry in the heatmap. From the results, we can see that the six samples are precisely clustered into two groups (Figure 6 and Figure S1 in the Supplementary Materials).

**Figure 7:**
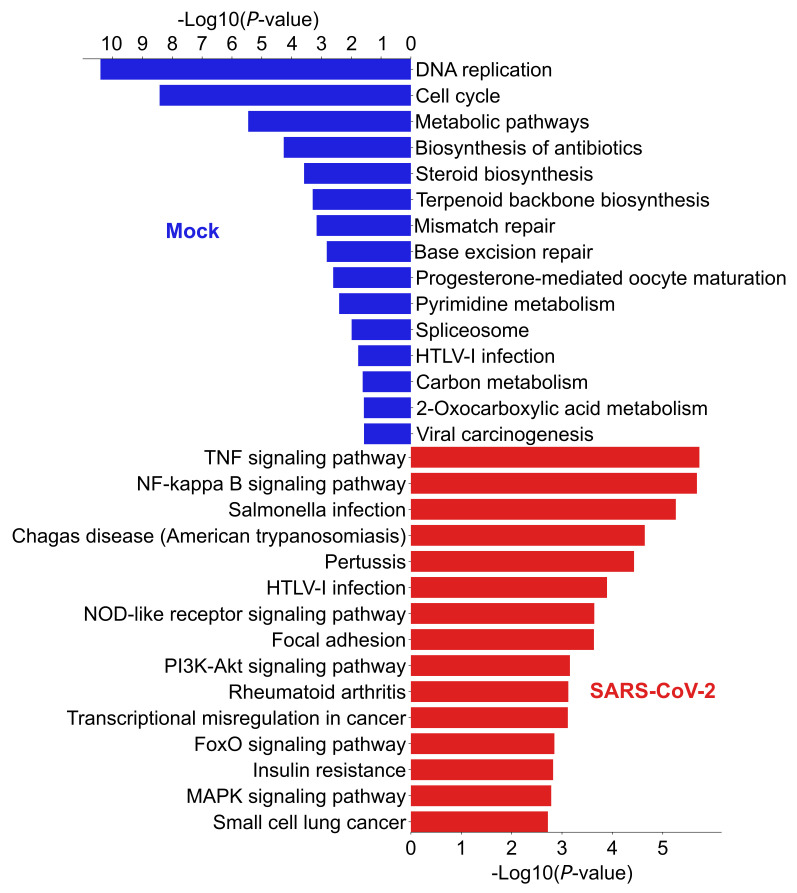
Differentially expressed transcripts enriched KEGG pathways. The blue and red bar charts show the pathways enriched by the up-regulated and down-regulated transcripts in mock-treated samples over SARS-CoV-2 infected samples, respectively.

Table 3 shows two differentially expressed genes that are related to SARS-CoV-2 pathogenesis and prognosis. Figure 7 and Figure S2 in the Supplementary Materials show the enriched KEGG pathways by the differentially expressed transcripts and genes, respectively. In each figure, the top bar plot represents the pathways enriched by the transcripts or genes that are highly expressed in mock-treated cells, and the bottom bar plot represents the transcripts or genes that are highly expressed in SARS-CoV-2 infected cells. The enriched pathways by the gene expression and transcript expression analyses are similar. The KEGG pathway enrichment of the differentially expressed transcripts in the SARS-CoV-2 in Figure 7 shows an up-regulation in the pro-inflammatory pathways TNF-α and NF-kappa, confirming the finding that patients with SARS-CoV-2 were reported to have elevated levels of cytokines in blood and cytokine gene expression in peripheral blood mononuclear cells (PBMC) [32,33,34].

### 2.5. Comparison between Alternative Processing of Pre-mRNA and Differential Gene Expression

In addition to mRNA expression, post-transcriptional regulation of gene expression (i.e., AS and APA) plays a critical role in the control of cellular processes and affects phenotypic changes in biological features [35]. They may serve as a new layer of biomarker for drug development for COVID-19 therapeutics. Therefore, it is important to compare the genes that are differentially expressed and the genes that undergo alternative processing events.

**Figure 8:**
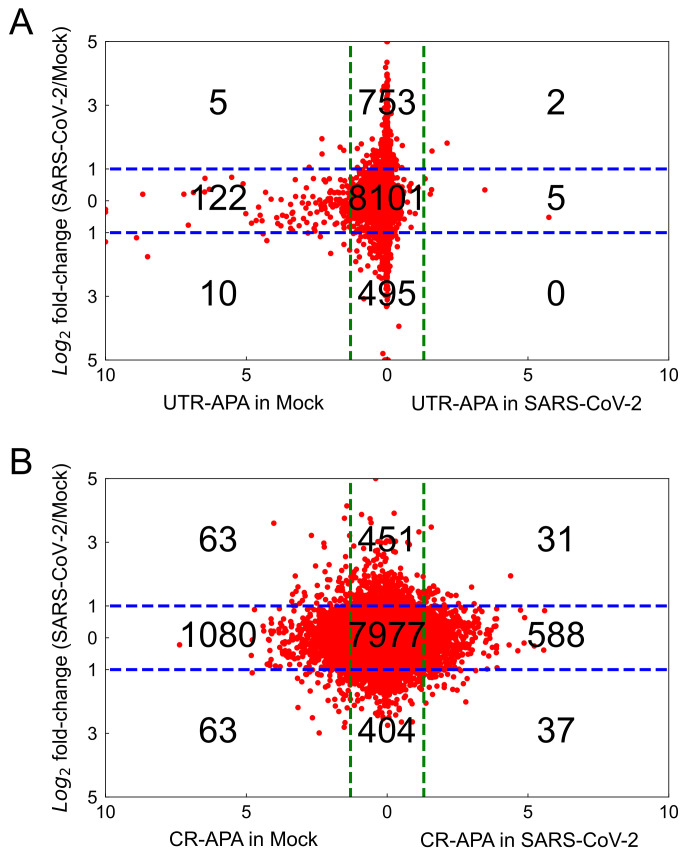
Scatter plot of APA and differential gene expression. Red dots represent the individual gene in the analysis. Horizontal blue-dashed lines represent the cutoff values for two-fold changes in differential gene expression. Vertical green-dashed lines represent the cutoff values for the $log_{10}$(*p*-value) of UTR-APA (**A**) determined by the Chi-squared test, and the $log_{10}$(*p*-value) of CR-APA (**B**) determined by the Student’s *t*-test.

The relationship between differential gene expression and the UTR-APA event was tested based on the APA events identified in Section 2.3.1 and the differentially expressed genes identified in Section 2.4. We combined the analyses of UTR-APA and differential gene expression, and the results are shown in Figure 8A. Each gene was plotted by fold changes in the differential gene expression (*y*-axis) and the significance of UTR-APA events (*x*-axis). The left three sections and the right three sections in Figure 8A show the 3${}^{\prime }$-UTR-truncated genes in mock-treated cells and SARS-CoV-2 infected cells, respectively. The top three sections and the bottom three sections represent the up-regulated and down-regulated genes in SARS-CoV-2 infected cells over mock treated cells, respectively. From the plot, we can see that a significant portion (>88%) of the genes showing UTR-APA events are not differentially expressed, indicating no strong correlation between the differential gene expression and the UTR-APA events in the SARS-CoV-2 infected cells. Similarly, more than 90% of the genes showing CR-APA events are not differentially expressed (Figure 8B). Therefore, the profiling of APA-based molecular signatures could provide additional predictive power and potentially lead to the findings of new target pathways for SARS-CoV-2 related translational research.

**Figure 9:**
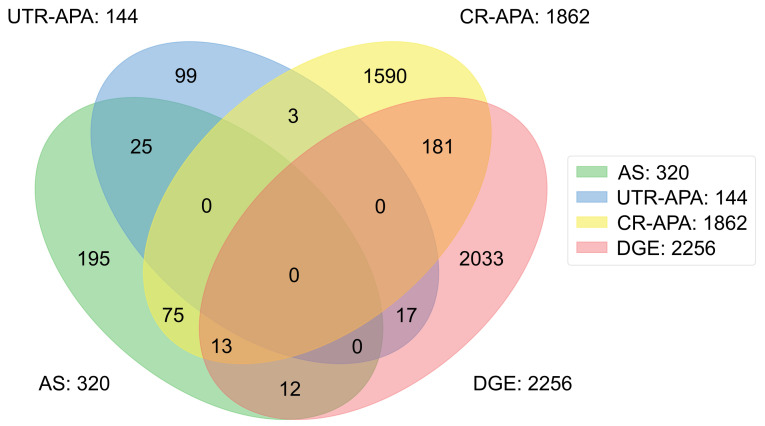
Four-set Venn diagram shows the overlapped genes in three different types of post-transcriptional regulations and differentially expressed genes (DEG).

To further investigate the relationship among all transcript variants identified in this study, a four-set Venn diagram is generated to illustrate the intersections of differentially expressed genes (DEG) and the genes with post-transcriptional regulation events (Figure 9). Both RefSeq and UCSC annotations were applied in the data analysis to make the experimental results comparable. The total number of events generated from alternative splicing, UTR-APA, CR-APA, and differentially expressed genes are 320, 144, 1862, and 2256, respectively. None of the genes were shown in all four categories. Though 2256 genes were detected as differentially expressed genes, only 25 of them have alternative splicing events and 17 of them have UTR-APA events. In the case of an alternative splicing event in a gene showing CR-APA, it has a higher chance of being considered as a CR-APA gene. Based on these observations, we found that 27.5% of genes with AS events show CR-APA. Overall, the results further confirm that post-transcriptional regulation profiles and differential gene expressions profiles provide distinctive molecular signatures for COVID-19 studies. In addition, the three different types of post-transcriptional regulation events barely overlap.

## 3. Discussion and Conclusions

At present, the whole world is suffering from the COVID-19 pandemic, but the molecular mechanisms of COVID-19 pathogenesis are not clear. To better understand the molecular basis of the disease, here we compare the effects of transcriptional and post-transcriptional events on SARS-CoV-2 infection in lung cancer cell lines. From these analyses, we were able to identify distinct molecular signatures that were not otherwise found by traditional differential gene expression analysis. These novel molecular signatures may contribute to COVID-19 pathobiology. The results from the data analyses demonstrate that several transcriptional and post-transcriptional signatures identified in this study are highly correlated with SARS-CoV-2 pathogenesis and prognosis. These signatures are enriched in diverse biological pathways that were not detected in the original study [1], which only focused on differential gene expression analysis. These pathways may lead to the discovery of new molecular mechanisms of SARS-CoV-2 pathogenesis. In addition, post-transcriptional gene regulations provide additional molecular signatures for COVID-19 therapeutic targets compared to the transcriptional signatures of COVID-19 patients. The latest COVID-19 study [10] has shown that the SARS-CoV-2 NSP16 protein suppresses mRNA splicing, leading to intron retention in host genes. In our data analyses, we only identified 16 intron retention events in the SARS-CoV-2 infected samples. This might be due to the fact that our custom-developed AS-Quant pipeline detects intron retention based on the annotations [26]. Thus, more cases for intron retention could be discovered by future pipelines, which will be important to identify non-canonical peptides produced from the translation of intron retained transcripts.

While our findings are confined at the transcriptome level, further investigations on the changes of proteome and post-translational modifications in the proteome are warranted to comprehensively dissect the mechanistic aspect of COVID-19 pathobiology. In our pathway enrichment analysis, genes in the ribosome pathway are up-regulated in the SARS-CoV-2 infected cells, suggesting that there may be an up-regulation of protein synthesis. Recent quantitative proteomics studies have suggested that time-dependent proteome changes in the post-infection of SARS-Cov-2 [36,37]. Studies have reported that in an early stage of infection (less than 24 h), a proteome change occurs bi-directionally (some up-regulated and some down-regulated). However, at a later stage of infection (greater than 24 h), there is an overall down-regulation of the global protein abundance. However, proteins that displayed differential phosphorylation and ubiquitination did not change the overall protein abundance. Nonetheless, 17.0%, 20.1%, and 16.4% of the genes that contain AS, UTR-APA, and CR-APA events are reported to be differentially expressed in their protein levels [37]. In addition, both our post-transcriptional study and the proteomics studies have consistently identified the activation of the MAPK signaling pathway in SARS-CoV-2 infected cells. These findings suggest the importance of using a multi-omics approach in resolving detailed molecular and cellular signatures of COVID-19.

## 4. Materials and Methods

In this section, we first introduce the pipelines and strategies to detect the alternative splicing and alternative polyadenylation events between two biological conditions in Section 4.1 and Section 4.2, respectively. Then, we explain the method to determine the differentially expressed transcripts and genes in Section 4.3. Finally, we provide an overview of the gene set enrichment analysis on the significant events in Section 4.4.

### 4.1. Detection of Alternative Splicing Events

To analyze the alternative splicing events between mock-treated and infected with SARS-CoV-2 samples with RNA-seq data, we used the computational pipeline AS-Quant [26]. Based on the read coverage files generated by SAMtools, all the potential splicing events were categorized into five major types of alternative splicing, as shown in Figure 1A. For each spliced exon, the average read coverage (*n*) is calculated by the read count on that exon ($r_{e}$) divided by the read length ($l_{e}$) in AS-Quant:

(1) $$n=\frac{r_{e}}{l_{e}}$$

The average read coverage for all other exons of that gene (*N*) is calculated in the same way, by dividing the total reads ($r_{g}$) by the gene length ($l_{g}$). Next, the ratio difference between two biological conditions is calculated based on the following equation:

(2) $$\frac{n_{1}}{N_{1}}-\frac{n_{2}}{N_{2}},$$

where 1 and 2 represent the different conditions. The most significant events are reported according to the *p*-value generated by a canonical 2 × 2 Chi-squared test. The topmost events are then illustrated by read coverage plots to give a visual representation (e.g., Figure 2B).

### 4.2. Detection of APA Events

#### 4.2.1. UTR-APA

To identify the 3*’*-UTR-APA events between mock-treated or SARS-CoV-2 infected samples with RNA-seq data, we applied the computational tool APA-Scan [27]. APA-Scan utilizes either predicted or experimentally validated polyadenylation signals as a reference for 3*’*-UTR polyadenylation sites. It also calculates the amount of full-length and truncated 3*’*-UTR transcripts with the RNA-seq data.

The canonical polyadenylation signals (PAS) AATAAA and ATTAAA in the distal 3*’*-UTR region were considered as the tentative locations of cleavage sites. Then, the APA-events were evaluated by comparing the RNA-seq reads coverage upstream and downstream of the candidate cleavage site between the samples in two different biological conditions. Specifically, the average read coverage for upstream ($M_{1}$ and $M_{2}$ for two biological conditions) and downstream ($m_{1}$ and $m_{2}$) of the candidate cleavage site were calculated. Next, the ratio difference between two conditions was determined by the following equation:

(3) $$\frac{m_{1}}{M_{1}}-\frac{m_{2}}{M_{2}}.$$

The 2 × 2 Chi-square test was then applied to assess the significance of the candidate events. The read coverage plots of the significant events were reported according to the user-defined *p*-value cutoff (e.g., Figure 3).

#### 4.2.2. CR-APA

To identify and quantify dynamic CR-APA events, the protein-coding transcripts of a gene were categorized as either full-length or truncated transcripts based on the NCBI human hg38 RefSeq [28] or UCSC annotations [38]. A transcript is categorized as a truncated transcript if it satisfies the following conditions: (1) its coding end is not the maximum coding end in the gene, and (2) its transcript end is not the maximum transcript end in the gene; otherwise, the transcript is a full-length transcript in the gene. The CR-truncation ratio $R_{CR}$ that quantifies the CR-APA event is defined as,

(4) $$R_{CR}=E_{CR}/E_{ALL}$$

where $E_{CR}$ is the summation of expressions of all truncated transcripts in the gene, $E_{ALL}$ denotes gene expression, and $R_{CR}\in \left[0,1\right]$. The Student’s *t*-test is then applied to the CR-truncation ratios to determine the significant CR-APA events between the two groups of samples.

### 4.3. Differential Gene/Transcript Expressions

Differential gene expression analysis has been widely used to identify transcriptomic signatures that define phenotypic changes in biology. In this work, Kallisto [12] was applied to perform the quantification of RNA-seq data for COVID-19 samples. After filtering out lowly expressed genes (TPM < 1), the $log_{2}$ fold change of transcript or gene expression was calculated between two groups of samples. The transcripts or genes with a $log_{2}$ fold change larger than 1 or smaller than −1, and with a Student’s *t*-test *p*-value < 0.05 were considered as significantly differentially expressed.

### 4.4. Enriched Pathway Analysis

After identifying the AS events, APA events, and differentially expressed genes/transcripts in each biological condition, the events and genes which are over-represented in GO (Gene Ontology) terms and KEGG (Kyoto Encyclopedia of Genes and Genomes) pathways were determined by DAVID Bioinformatics Resources [39]. These enriched functional profiles and molecular interactions could help us better understand the underlying biological processes in COVID-19. The KEGG pathway is a collection of pathway-maps representing molecular level information, including metabolism, genetic information processing, environmental information processing, cellular processes, organismal systems, human diseases, and drug development. The Gene Ontology (GO) terms define the biological domain with respect to three aspects: molecular function, cellular component, and biological process.

## Figures and Tables

**Table 1:** Number of detected significant splicing events in each category between SARS-CoV-2 infected and mock-treated cells.

Sample	SE	RI	MXE	A3SS	A5SS
Mock	51	16	7	13	6
SARS-CoV-2	126	30	19	59	100

**Table 2:** Top 10 significant splicing events between A549 mock-treated and SARS-CoV-2 infected cells.

Gene Name	Chr	Start	End	*p*-Value	FDR	Ratio Difference
TPT1	chr13	45,914,846	45,914,920	5.81 × 10${}^{-18}$	7.5 × 10${}^{-14}$	−0.074
C6ORF48	chr6	3,118,955	3,119,049	9.21 × 10${}^{-15}$	5.94 × 10${}^{-11}$	−0.113
FKBP1A	chr20	1,373,477	1,373,525	2.25 × 10${}^{-14}$	9.67 × 10${}^{-11}$	−0.168
PPIA	chr7	44,838,346	44,838,413	1.63 × 10${}^{-12}$	5.25 × 10${}^{-9}$	0.149
HNRNPA1	chr12	54,676,862	54,677,018	1.26 × 10${}^{-11}$	3.26 × 10${}^{-8}$	−0.248
RPS24	chr10	79,799,961	79,799,983	5.25 × 10${}^{-11}$	1.13 × 10${}^{-7}$	0.122
RPS9	chr19	54,710,420	54,710,592	4.59 × 10${}^{-10}$	8.46 × 10${}^{-7}$	0.117
SRSF2	chr17	74,731,853	74,731,957	1.24 × 10${}^{-7}$	1.78 × 10${}^{-4}$	0.172
CA12	chr15	63,638,728	63,638,908	1.66 × 10${}^{-7}$	1.99 × 10${}^{-4}$	−0.103
RPLP1	chr15	69,745,985	69,746,060	1.69 × 10${}^{-7}$	1.99 × 10${}^{-4}$	0.040

**Table 3:** Literature review of identified genes with transcript variants and their implication on SARS-CoV-2 pathogenesis and prognosis.

Category	Gene	Ref.	Description
Alternative Splicing	BTF3	[15]	Interacts with the NSP10 CoV protein, which is involved in the pathological function of SARS-CoV in cells.
	FKBP1A	[16]	FKBP1A causes immunosuppression and is required by CoV for viral growth.
	G3BP1	[17]	SARS-CoV-2 N protein undergoes liquid–liquid phase separation, which serves as a scaffold for virus replication and assembly, through its N-terminal intrinsically disordered region (IDR) with G3BP1.
UTR-APA	ANXA2	[18]	The upregulation of expression of annexin A2 (ANXA2) by SARS-associated cytokines and the cross-reactivity of anti-SARS-CoV S2 antibodies to annexin A2 may have implications in SARS disease pathogenesis.
	CAV1	[19]	Coronaviruses enter cells via the CAV1 dependent pathway.
	TMEM97	[20]	TMEM97 forms a complex with ACE2 and modulates its ability to internalize the SARS-CoV-2.
CR-APA	CTSC	[21]	CTSC activates the elastase-related neutrophil proteases mediated tissue degradation in which it diffuses the alveolar inflammation in acute respiratory distress syndrome.
	RHOA	[22]	Activation of RhoA GTPase and its downstream effector, Rho kinase (ROCK), contributes to a burst in inflammatory features, immune cell migration, apoptosis, coagulation, contraction, and cell adhesion in pulmonary endothelial cells, leading to endothelium barrier dysfunction and edema as hallmarks of lung injury.
	CANX	[23]	Calnexin (CANX) strictly monitors the maturation of the CoV S protein by its direct binding.
Differential Expression	BCL2A1	[24]	Pro-survival gene mostly present in adult genes, if downregulated, promotes apoptosis in lung tissue.
	SKP2	[25]	SKP2 attenuates autophagy through Beclin1-ubiquitination and allowing for the replication of coronaviruses.

**Table 4:** Top 10 significant alternative polyadenylation events in the 3${}^{\prime }$-UTR region (UTR-APA) between A549 mock-treated and SARS-CoV-2 infected cells. The ‘Position’ column refers to the potential cleavage site for the UTR-APA.

Gene Name	Chr	Position	*p*-Value	FDR	Ratio Difference
ACTN4	chr19	38,730,184	6.07 × 10${}^{-14}$	5.71 × 10${}^{-10}$	0.144
ALDH1A1	chr9	72,900,986	1.34 × 10${}^{-12}$	6.30 × 10${}^{-9}$	0.054
S100A6	chr1	153,534,690	2.73 × 10${}^{-11}$	8.56 × 10${}^{-8}$	0.045
HNRNPA2B1	chr7	26,191,861	1.26 × 10${}^{-9}$	2.96 × 10${}^{-6}$	0.088
PMEPA1	chr20	57,651,579	2.10 × 10${}^{-9}$	3.95 × 10${}^{-6}$	0.153
ACAT2	chr6	159,779,033	3.11 × 10${}^{-9}$	4.88 × 10${}^{-6}$	0.273
ARL4C	chr2	234,495,151	6.05 × 10${}^{-8}$	8.14 × 10${}^{-5}$	0.231
H3F3A	chr1	226,071,775	8.70 × 10${}^{-8}$	1.02 × 10${}^{-4}$	0.189
RPL15	chr3	23,919,537	1.32 × 10${}^{-7}$	1.38 × 10${}^{-4}$	0.041
MYH9	chr22	36,281,660	3.29 × 10${}^{-7}$	2.91 × 10${}^{-4}$	0.177

**Table 5:** Top 10 significant alternative polyadenylation events in the coding region (CR-APA) between A549 mock-treated and SARS-CoV-2 infected cells.

Gene	Chr	Truncated Position	*p*-Value	FDR	Ratio Difference
GLRX5	chr14	95,544,557	2.76 × 10${}^{-6}$	6.12 × 10${}^{-3}$	0.361
UBXN6	chr19	4,445,009	5.98 × 10${}^{-6}$	1.14 × 10${}^{-2}$	0.020
SLC3A2	chr11	62,881,290	1.31 × 10${}^{-5}$	2.16 × 10${}^{-2}$	0.256
ARIH2	chr3	48,928,674	1.37 × 10${}^{-5}$	1.18 × 10${}^{-2}$	0.071
DVL3	chr3	184,164,517	1.50 × 10${}^{-5}$	2.16 × 10${}^{-2}$	−0.269
DEF8	chr16	89,959,366	1.62 × 10${}^{-5}$	1.18 × 10${}^{-2}$	−0.232
SIAH2	chr3	150,742,487	1.62 × 10${}^{-5}$	2.16 × 10${}^{-2}$	−0.362
SNHG7	chr9	136,724,659	1.84 × 10${}^{-5}$	2.16 × 10${}^{-2}$	0.020
LTBP2	chr14	74,506,705	1.94 × 10${}^{-5}$	2.16 × 10${}^{-2}$	−0.248
S100A16	chr1	153,606,974	2.29 × 10${}^{-5}$	2.35 × 10${}^{-2}$	0.200

## Data Availability

The dataset GSE147507 used in this study is available at the NCBI GEO Database (accessed on 15 April 2020).

## Supplementary Materials

The following are available at https://www.mdpi.com/article/10.3390/ijms22189684/s1.

## Abbreviations

RT-PCR	Real-Time Polymerase Chain Reaction
CR-APA	Coding Region Alternative PolyAdenylation
UTR-APA	UnTranslated Region Alternative PolyAdenylation
AS-Quant	Alternative Splicing Quantitation
APA-Scan	Alternative Polyadenylation Scan
PAS	PolyAdenylation Signal
DEG	Differentially Expressed Genes
MAPK	Mitogen-Activated Protein Kinase
GEO	Gene Expression Omnibus
GO	Gene Ontology
KEGG	Kyoto Encyclopedia of Genes and Genomes
