# Supplementary material for: Computational Methods to Study Human Transcript Variants in COVID-19 Infected Lung Cancer Cells
Source: Int J Mol Sci. 2021 Sep 7;22(18):9684. doi: 10.3390/ijms22189684 (PMC8464664; doi:10.3390/ijms22189684)
Supplement: Supplementary file 1 [file ijms-22-09684-s001.zip › ijms-1302838-supplementary.pdf]

Computational Methods to Study Human Transcript Variants in COVID-19 Infected Lung Cancer Cells

September 7, 2021

1 Data summarization

Sample Name	Alignment Rate (hg38)	Alignment Rate (hg19)	Description
GSM4432384	86.20%	83.20%	Series2_A549_Mock_1
GSM4432385	84.60%	83.10%	Series2_A549_Mock_2
GSM4432386	80.50%	78.60%	Series2_A549_Mock_3
GSM4432387	86.10%	84.80%	Series2_A549_SARS-CoV-2_1
GSM4432388	80.60%	78.40%	Series2_A549_SARS-CoV-2_2
GSM4432389	84.70%	82.90%	Series2_A549_SARS-CoV-2_3
GSM4462336	83.30%	81.50%	Series5_A549_Mock_1
GSM4462337	81.50%	80.10%	Series5_A549_Mock_2
GSM4462338	82.50%	80.90%	Series5_A549_Mock_3
GSM4462339	83.20%	81.40%	Series5_A549_SARS-CoV-2_1
GSM4462340	82.90%	81.40%	Series5_A549_SARS-CoV-2_2
GSM4462341	84.50%	83.30%	Series5_A549_SARS-CoV-2_3
GSM4462348	83.40%	81.50%	Series7_Calu3_Mock_1
GSM4462349	82.20%	78.20%	Series7_Calu3_Mock_2
GSM4462350	83.60%	82.10%	Series7_Calu3_Mock_3
GSM4462351	73.00%	71.50%	Series7_Calu3_SARS-CoV-2_1
GSM4462352	68.60%	66.80%	Series7_Calu3_SARS-CoV-2_2
GSM4462353	69.80%	63.10%	Series7_Calu3_SARS-CoV-2_3

Table S1: Summary of COVID-19 samples (GSE147507).

2 Alternative splicing

Gene	Chromosome	Start	End	p-value	FDR	Ratio Difference	AS type
TPT1	chr13	45914846	45914920	9.94E-05	1.0	-0.0295	SE
C17ORF76-AS1	chr17	16342841	16343017	0.00097	1.0	0.0871	SE
RPS24	chr10	79799961	79799983	0.0023	1.0	-0.0701	SE
NT5C2	chr10	104865114	104865138	0.0058	1.0	0.1793	SE
MYL6	chr12	56554409	56554454	0.013	1.0	-0.0364	SE
RPS3	chr11	75111737	75111868	0.023	1.0	0.0121	SE
TRIM35	chr8	27147672	27147695	0.025	1.0	0.4094	SE
SAE1	chr19	47656154	47656297	0.0407	1.0	0.0719	SE
DCTN2	chr12	57932301	57932307	0.0187	1.0	-0.0789	MXE
C17ORF76-AS1	chr17	16342841	16342893	5.95E-20	2.61E-16	0.2377	A3SS
C17ORF76-AS1	chr17	16342841	16342972	3.639E-07	0.000798	0.1302	A3SS
C17ORF76-AS1	chr17	16342894	16342972	0.0219	1.0	0.0641	A3SS
FBL	chr19	40331255	40331427	0.000257	0.7239	0.0923	A5SS
PFDN6	chr6	33257398	33257405	0.0066	1.0	0.1131	A5SS
ACTG1	chr17	79479639	79479892	0.0459	1.0	0.0154	A5SS

Table S2: Significant alternative splicing events detected between Series2 A549 mock treated and Series2 A549 SARS-CoV-2 infected cells.

Gene	Chromosome	Start	End	p-value	FDR	Ratio Difference	AS type
HMGA1	chr6	34204980	34205094	3.43E-37	4.18E-33	-0.218	SE
TNFRSF12A	chr16	3071215	3071320	2.54E-16	1.55E-12	-0.158	SE
RPS9	chr19	54710420	54710592	4.24E-11	1.72E-07	0.180	SE
TPT1	chr13	45914846	45914920	6.98E-10	2.13E-06	-0.076	SE
GRAMD1A	chr19	35500326	35500347	9.037E-08	0.0002	-0.590	SE
PLAU	chr10	75671798	75671826	2.326E-06	0.0047	-0.084	SE
HNRNPC	chr14	21730759	21730927	4.108E-06	0.0071	0.236	SE
BAG6	chr6	2893732	2893876	1.39E-05	0.0211	0.144	SE
HN1	chr17	73142760	73142779	4.66E-05	0.0567	-0.151	SE
SAT1	chrX	23802410	23802520	9.74E-05	0.1079	0.122	SE
IGFBP3	chr7	45960336	45960871	1.604E-21	2.32E-18	-0.163	RI
HMGA1	chr6	34204649	34205094	4.096E-13	2.96E-10	-0.112	RI
JUP	chr17	39910858	39912147	9.31E-06	0.0045	0.083	RI
ACADVL	chr17	7123149	7123516	0.00028	0.105	-0.204	RI
HLA-A	chr6	29910533	29911320	0.0011	0.276	-0.131	RI
SLC25A3	chr12	98987402	98987913	0.0011	0.276	-0.127	RI
SRSF2	chr17	74730196	74732546	0.0131	1.0	0.148	RI
CD164	chr6	109687716	109690220	0.0158	1.0	0.108	RI
CD59	chr11	33743924	33744273	0.016	1.0	-0.087	RI
ATF4	chr22	39916568	39917676	0.016	1.0	-0.081	RI
PLAU	chr10	75671798	75671826	2.326E-06	0.005	-0.084	MXE
CPNE1	chr20	34243123	34243266	0.0003	0.297	0.203	MXE
H2AFV	chr7	44882875	44882953	0.0068	1.0	-0.139	MXE
BID	chr22	18232870	18232940	0.007	1.0	-0.161	MXE
TPM2	chr9	35684728	35684804	0.0106	1.0	0.142	MXE
TPM1	chr15	63353911	63353987	0.0115	1.0	0.089	MXE
CKLF-CMTM1	chr16	66592092	66592251	0.019	1.0	0.096	MXE
TRA2A	chr7	23561750	23562051	0.027	1.0	0.211	MXE
CKLF	chr16	66592092	66592251	0.028	1.0	0.089	MXE
TRA2A	chr7	23561739	23562051	0.0299	1.0	0.208	MXE
RPL22L1	chr3	170585924	170585990	1.144E-08	4.84E-05	0.305	A3SS
ORMDL3	chr17	38080474	38080478	3.0265E-08	6.4E-05	-0.1005	A3SS
9 THRA	chr17	38249272	38249388	0.00018	0.254	0.247	A3SS
TNIP1	chr5	150444689	150444692	0.0008	0.890	-0.133	A3SS
RPL17	chr18	47017955	47018203	0.001	0.890	0.150	A3SS
ERBB2	chr17	37850933	37851239	0.002	1.0	0.017	A3SS
PVR	chr19	45150494	45150570	0.005	1.0	-0.197	A3SS
RPLP0	chr12	120638635	120638694	0.01	1.0	-0.056	A3SS
ATP2C1	chr3	130613433	130613550	0.015	1.0	-0.136	A3SS
COPS2	chr15	49429635	49429655	0.021	1.0	0.284	A3SS
IGFBP3	chr7	45960317	45960871	6.29E-21	1.74E-17	-0.158	A5SS
HMGA1	chr6	34204739	34205094	3.36E-14	4.64E-11	-0.122	A5SS
KRT8	chr12	53343238	53343650	1.92E-11	1.77E-08	0.066	A5SS
C18ORF32	chr18	47013411	47013644	8E-05	0.047	-0.272	A5SS
RBM12	chr20	34252680	34252878	8.51E-05	0.047	-0.207	A5SS
IFI6	chr1	27995742	27995857	0.00016	0.073	0.086	A5SS
IFI6	chr1	27995730	27995857	0.00022	0.086	0.086	A5SS
RPL17	chr18	47018626	47018834	0.00029	0.101	-0.164	A5SS
CNBP	chr3	128890475	128890614	0.00132	0.379	-0.139	A5SS
RPS14	chr5	149829047	149829319	0.0015	0.379	-0.033	A5SS

Table S3: Significant alternative splicing events detected between Series7 Calu3 mock treated and Series7 Calu3 SARS-CoV-2 infected cells. The top 10 events for each AS type are reported.

3 Alternative polyadenylation

3.1 CR-APA

Gene	Chromosome	Truncated Position	p-value	FDR	Ratio Difference
SLC26A10	chr12	57626140	1.028E-05	6.6607E-02	-0.4349989
MX1	chr21	41423277	5.9603E-05	1.1394E-01	-0.5629043
COLCA1	chr11	111293388	6.1547E-05	1.1394E-01	0.4185045
CEP55	chr10	93519111	8.0216E-05	1.2994E-01	0.05407685
HADH	chr2	26191334	9.9116E-05	1.4272E-01	0.05499882
PCDHGA7	chr5	141386000	0.00015904	2.0610E-01	-0.4670165
CCDC39	chr3	180614587	0.00023673	2.3598E-01	-0.2736101
MSRA	chr8	10250917	0.00053088	3.1288E-01	-0.1333415
BTBD11	chr12	107650897	0.00064725	3.1288E-01	-0.1024467
CDK18	chr1	205526835	0.00066146	3.1288E-01	-0.1983633

Table S4: Top 10 significant alternative polyadenylation events in the coding region (CR-APA) between Series2 A549 mock treated and Series2 A549 SARS-CoV-2 infected cells.

Gene	Chromosome	Truncated Position	p-value	FDR	Ratio Difference
FAM228B	chr2	24080955	1.2312E-06	1.4855E-02	0.30397228
TMEM242	chr6	157318180	9.7391E-06	3.0997E-02	0.27816323
PLAUR	chr19	43648570	1.7915E-05	2.5027E-02	0.12882853
NEDD4	chr15	55862982	2.0103E-05	3.8092E-02	-0.3406388
PMPCA	chr9	136412504	3.967E-05	5.0503E-02	0.27429435
RIPK2	chr8	89762858	5.0364E-05	5.8289E-02	-0.1270158
TMEM125	chr1	43270820	6.5721E-05	6.4361E-02	0.21102247
FAM3A	chrX	154506710	0.00012337	1.0537E-01	0.22407826
CCDC88C	chr14	91278140	0.00012415	1.0537E-01	0.15202499
DVL3	chr3	184164517	0.00013498	1.0740E-01	-0.2263373

Table S5: Top 10 significant alternative polyadenylation events in the coding region (CR-APA) between Series7 Calu3 mock treated and Series7 Calu3 SARS-CoV-2 infected cells.

3.2 UTR-APA

Gene	Chromosome	Truncated Position	p-value	FDR	Ratio Difference
SPP1	chr4	87982898	3.42E-06	0.0323	0.0626
ACAT2	chr6	159779033	0.000133	0.6302	0.2001
TCP1	chr6	159778972	0.00037	0.9208	-0.1863
SDC4	chr20	45325816	0.00038	0.9207	-0.1049
PABPC1	chr8	100703253	0.000616	1.0	0.2434
HNRNPA2B1	chr7	26191861	0.00089	1.0	0.0750
TRMT112	chr11	64316660	0.00098	1.0	0.1553
ANXA2	chr15	60347286	0.00405	1.0	0.0069
RPN1	chr3	128620181	0.0068	1.0	0.0751
OAZ1	chr19	2273313	0.016082353	1.0	0.0371

Table S6: Top 10 significant alternative polyadenylation events in the 3'-UTR region (UTR-APA) between Series7 Calu3 mock treated and Series7 Calu3 SARS-CoV-2 infected cells.

4 Differential transcript expression

Gene	Transcript	Series2_A549_Mock	Series2_A549_SARS-CoV-2	log2(Series2_A549_Mock/ Series2_A549_SARS-CoV-2)	*p*-value	FDR
HRAS	NM_001130442	5.837263	1.15274	2.340225	0.014258	0.700417
PSRC1	NM_032636	6.665747	1.57116	2.084936	0.031601	0.735119
CREB3L4	NM_001255978	5.933497	1.582624	1.906564	0.028207	0.735119
RAB5B	NM_001252036	11.42258	3.526733	1.695484	0.039039	0.735119
ALKBH2	NM_001001655	7.480163	2.894477	1.369767	0.003762	0.557131
IFI27	NM_001288956	0	5.483537	-inf	0.017906	0.730942
NDUFC1	NM_001184990	0	5.31663	-inf	0.025707	0.735119
MX1	NM_002462	0.096261	16.56603	-7.42706	3.7E-05	0.118172
IFI6	NM_022872	2.211427	45.5787	-4.36531	0.000683	0.349558
IFI6	NM_002038	10.94062	202.4793	-4.21001	0.000106	0.171799

Table S7: Top 10 differentially expressed transcripts between Series2 A549 mock treated and Series2 A549 SARS-CoV-2 infected cells.

Gene	Transcript	Series7_Calu3_Mock	Series7_Calu3_SARS-CoV-2	log2(Series7_Calu3_Mock/ Series7_Calu3_SARS-CoV-2)	*p*-value	FDR
SLC35A2	NM_001032289	8.636033	1.77E-05	18.89679	0.000247	0.025217
NTMT1	NM_001286800	5.31679	0.327757	4.019856	0.015406	0.168671
IDH2	NM_001290114	5.390877	0.358013	3.912435	0.010342	0.134916
EFNA4	NM_182689	5.626903	0.384437	3.871523	0.007106	0.109731
WDR13	NM_001347219	5.714217	0.39299	3.86199	0.019173	0.189557
CSF3	NM_172219	0	5.28365	-inf	2.02E-05	0.013314
CCL5	NM_001278736	0	21.01137	-inf	0.001281	0.049073
IFIT1	NM_001270928	0	7.193503	-inf	0.000982	0.044446
TPM1	NM_001018004	0	13.43094	-inf	0.030602	0.245658
IFNL3	NM_172139	0	36.82477	-inf	0.02339	0.211872

Table S8: Top 10 differentially expressed transcripts between Series7 Calu3 mock treated and Series7 Calu3 SARS-CoV-2 infected cells.

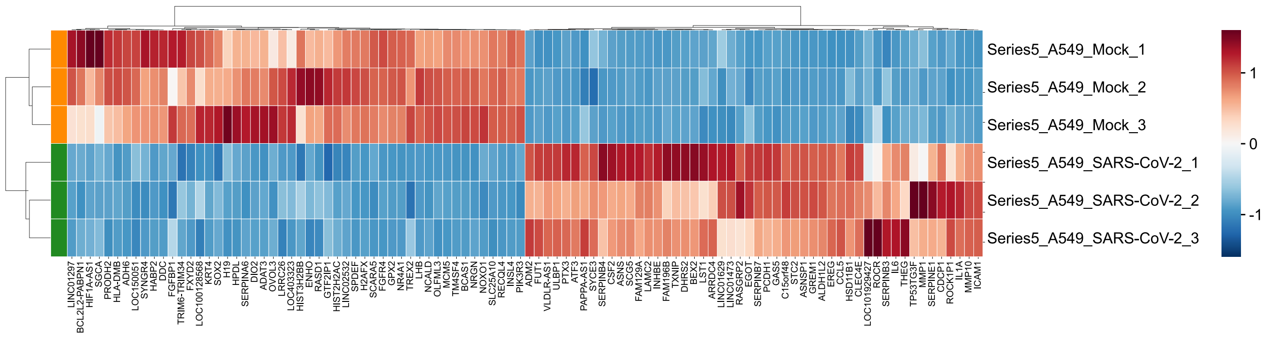

Figure S1: COVID-19 A549 cell lines are clustered by top 100 gene markers detected by differential gene expression analysis.

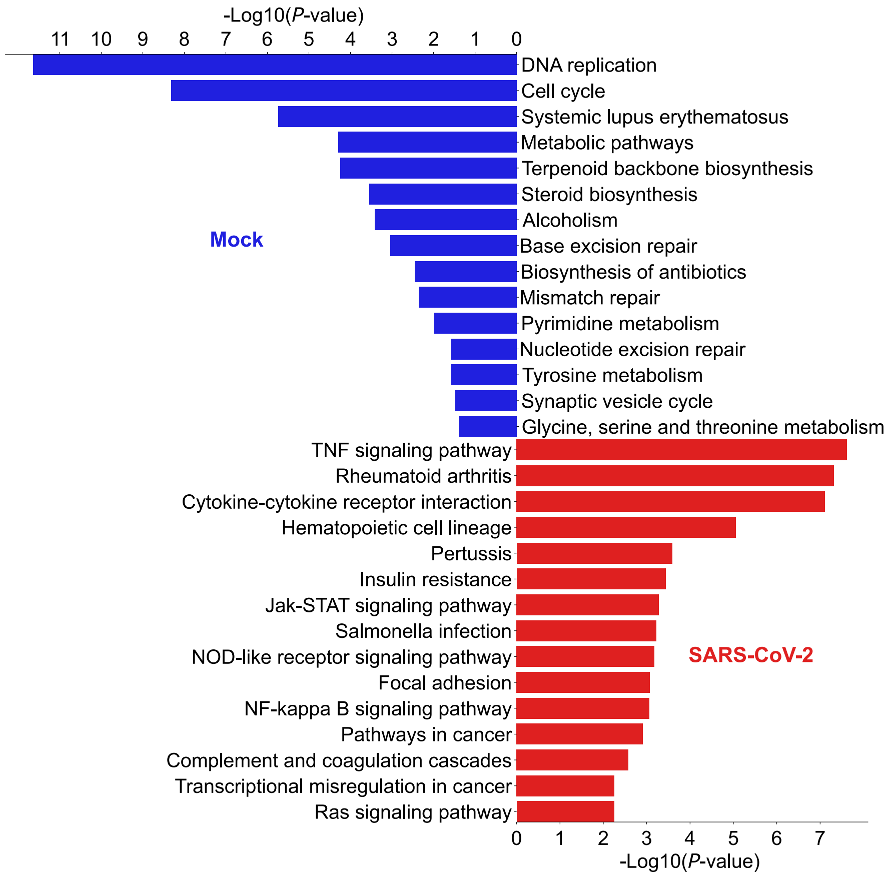

Figure S2: Differentially expressed genes enriched KEGG pathways. The blue and red bar charts show the pathways enriched by the up-regulated and down-regulated genes in mock treated samples over SARS-CoV-2 infected samples, respectively.
